# CLDN18 and FGFR2 splicing variants are emerging tumor-associated antigens for cancer therapy

**DOI:** 10.3389/fmmed.2026.1927389

**Published:** 2026-09-25

**Authors:** Masuko Katoh, Masaru Katoh

**Affiliations:** Department of Global Network, M & M Precision Medicine, Tokyo, Japan

**Keywords:** clinical immunohistochemistry, epithelial–mesenchymal plasticity, extrachromosomal DNA amplification, immune checkpoint inhibitor, immunosuppressive tumor microenvironment, intertumor heterogeneity, receptor tyrosine kinase, tight junction

## Abstract

Alternative splicing and alternative promoters generate splicing variants to expand proteomic and transcriptional diversity to orchestrate physiological activities and dictate tumorigenesis. Splicing variants are attractive tumor-associated antigens (TAAs) for the clinical development of cancer therapies. Here, recent progress in claudin-18 isoform 2 (CLDN18.2)- and fibroblast growth factor receptor 2 isoform b (FGFR2b)-targeted therapeutics is summarized, and perspectives in this field are discussed. Zolbetuximab and bemarituzumab are first-generation monoclonal antibodies (mAbs) targeting CLDN18.2 and FGFR2b, respectively, while next-generation biologics targeting these TAAs are emerging. Single-agent objective response rates of CLDN18.2-targeting mAb (omectatug), antibody–drug conjugates (ADCs, arcotatug tavatecan, BL-M05D1, garetatug rezetecan, JS107, sonesitatug vedotin and tecotabart vedotin), bispecific antibodies (emaretamig and givastomig) and chimeric antigen receptor T (CAR-T) cells (IMC002) were higher than that of zolbetuximab. Gastrointestinal toxicity, nausea and vomiting are common on-target/off-tumor adverse effects of CLDN18.2-targeted biologics, which are mitigated for arcotatug tavatecan and givastomig as a result of antibody engineering. Combination therapy of zolbetuximab plus chemotherapy is approved for the treatment of gastric and gastroesophageal junction cancer patients, while results of other CLDN18.2-targeting biologics in phase 3 clinical trials are anticipated with expectations for improved clinical activities and mitigated adverse events. Diagnostics detecting alternative isoforms and combination strategies using immune checkpoint inhibitors would address resistance mechanisms, such as epitope-losing isoform switching and immune evasion. Comparative studies on ADC monotherapy versus combination mAb-chemotherapy as well as the clinical development of bispecific ADCs and allogeneic CAR-T cells should further improve the clinical benefits of CLDN18.2- and FGFR2b-targeted therapies.

## 1 Introduction

Many transcript isoforms that contribute to proteomic and transcriptional diversity are generated via alternative splicing of context-dependent exons and the use of alternative promoters of distinct first exons, respectively, and regulate a variety of binary cell fates and processes: stemness versus differentiation; epithelial versus mesenchymal; proliferation versus senescence; survival versus apoptosis; and immune surveillance versus immune evasion (Bonnal et al., 2020; Bradley and Anczuków, 2023). Altered activity or expression of splicing regulators, such as ESRP1, ESRP2, SF3B1, SRSF2 and U2AF1, can globally dysregulate alternative splicing, while splice-site mutations in proto-oncogenes and tumor suppressor genes can locally dysregulate alternative splicing during multistage tumorigenesis. Isoforms can be targeted via small-molecules and splice-switching oligos preclinically (Stanley and Abdel-Wahab, 2022; Yan et al., 2025), while diverse biologics are in various stages of clinical development, with one potential benefit being reduced off-target effects by not targeting all isoforms from a gene (Katoh et al., 2024b; Kjer-Hansen et al., 2024). The most developed therapies target claudin-18 isoform 2 (CLDN18.2) and fibroblast growth factor receptor 2 isoform b (FGFR2b), which are representative tumor-associated splicing variants (meaning isoforms or encoded protein products hereafter), as cancer therapy (Figure 1).

**FIGURE 1:**
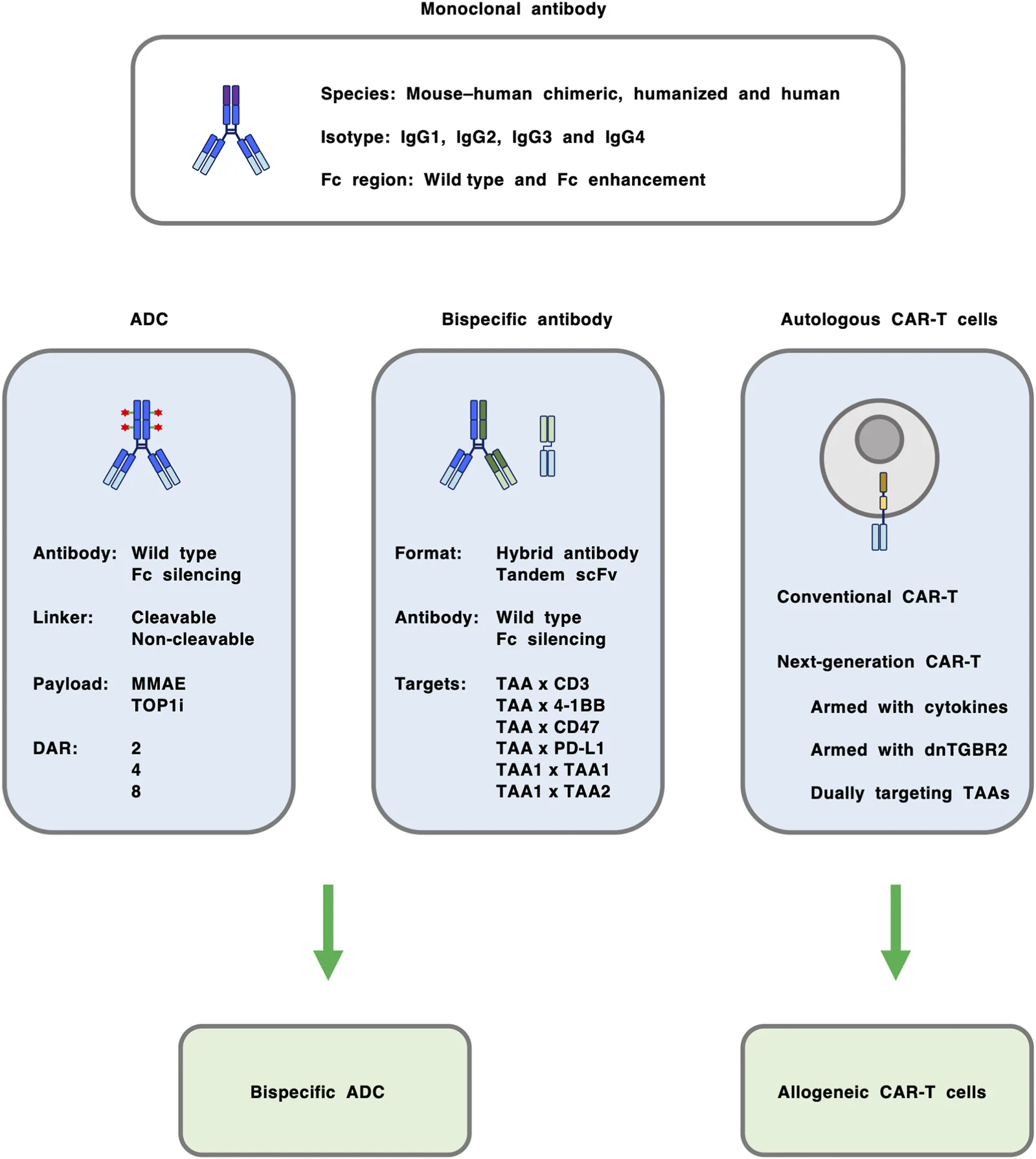
Modality of biologics. Monoclonal antibody (mAb), antibody–drug conjugate (ADC), bispecific antibody and chimeric antigen receptor T (CAR-T) cells are representative formats of biologics targeting tumor-associated antigens (TAAs). mAbs evolved from mouse–human chimeric antibody to human/humanized antibody with fragment crystallizable (Fc) enhancement, and are then applied for the generation of mAb derivatives, such as ADC, bispecific antibody and CAR-T cells. ADCs consist of mAb, linkers and cytotoxic payloads, such as monomethyl auristatin E (MMAE) and topoisomerase I inhibitor (TOP1i). Bispecific antibodies are classified into T-cell engagers (TAA x CD3), immune engagers (TAA x 4-1BB and TAA x CD47), dual epitope binders (TAA1 x TAA1) and dual antigen binders (TAA1 x TAA2). Fc silencing is introduced to some ADCs and bispecific antibodies to mitigate excessive antibody-dependent cellular cytotoxicity. Autologous CAR-T cells evolved from conventional CAR-T to next-generation CAR-T that are armed with cytokines or dominant-negative TGFβ receptor II (dnTGFBR2), or dually targeting TAAs. Bispecific ADCs and allogeneic CAR-T cells are anticipated to further improve clinical benefits of biologics for the treatment of cancer patients in the near future. IgG, immunoglobulin G; DAR, drug–antibody ratio; scFv, single-chain variable fragment.

Gastric cancer is the fifth most common malignancy worldwide, with approximately 970,000 new cases and 660,000 deaths in 2022 (Bray et al., 2024), and its risk factors include chronic gastritis associated with *Helicobacter pylori* infection (Malfertheiner et al., 2023), unfavorable lifestyles (consumption of alcohol and preserved foods) (Jin et al., 2020), germline mutations in the *CDH1* and *BRCA2* genes (Hansford et al., 2015) and single nucleotide polymorphisms in the *MUC1* and *PSCA* loci (Mocellin et al., 2015).

Gastric adenocarcinomas occur in the proximal cardia, gastric body and distal antrum, while gastroesophageal junction (GEJ) adenocarcinomas occur when the epicenter is no more than 2 cm proximal to the GEJ and extends into the esophagus. On the basis of similar genomic landscapes of gastric cancer and GEJ cancers [chromosomal instability subtype (51% versus 72%), genomically stable subtype (38% versus 20%), hypermutated subtype (6% versus 4%) and Epstein–Barr virus-positive subtype (5% versus 5%)], gastric and GEJ cancer are merged into the concept of G/GEJ cancer in clinical practice (Brezden-Masley et al., 2024; Shitara et al., 2025b).

Because CLDN18.2 and FGFR2b are overexpressed in subsets of G/GEJ cancers, therapeutics targeting CLDN18.2 or FGFR2b have been developed as investigational drugs. Zolbetuximab (IMAB362), an anti-CLDN18.2 mouse/human chimeric monoclonal antibody (mAb), in combination with conventional chemotherapies has been approved for the treatment of G/GEJ cancer patients by the US Food and Drug Administration (FDA), while other antibody-based drugs and/or cell-based therapeutics targeting CLDN18.2 (Katoh and Katoh, 2024; Nakayama et al., 2024) and FGFR2b (Katoh et al., 2024a; Wainberg et al., 2022) are in later-stage clinical trials. Here, recent progress in CLDN18.2- and FGFR2b-targeted therapies for G/GEJ cancer patients is overviewed, and future perspectives on splice variant-targeted therapies are discussed.

## 2 CLDN18.2-targeted therapies

The 24 human *claudin (CLDN)* genes encode 26 CLDN proteins at tight junctions that consist of four transmembrane domains, the N-terminal cytoplasmic region, two extracellular loops (ECL1 and ECL2), one cytoplasmic loop and the C-terminal cytoplasmic region (CTR) (Katoh and Katoh, 2024). ECLs and the CTR are involved in the homophilic/heterophilic interactions of CLDNs themselves and zonula occludens (ZO)-mediated tethering to actin filaments, respectively (Meoli and Günzel, 2023; Zihni et al., 2016). On the basis of these extracellular and cytoplasmic protein–protein interactions, CLDN proteins perform versatile physiological functions: (i) separation of apical and basolateral surfaces to establish apical–basal polarity in epithelial cells; (ii) regulation of paracellular permeability (barrier vs. channel) to maintain homeostasis in various organs; and (iii) indirect orchestration of Hippo–YAP/TAZ, PI3K-AKT and WNT/β-catenin signaling cascades via cell–cell adhesion networks to modulate cell fates and epithelial–mesenchymal plasticity (Katoh et al., 2025; Günzel et al., 2026; Saviano et al., 2026).

CLDN family members function as tumor promoters and/or tumor suppressors depending on tumor type, subtype, stage and experimental conditions in preclinical studies using cell lines and engineered mouse models (Li, 2021). Apart from debates about their functional consequences in preclinical model experiments, CLDN1, CLDN4, CLDN6 and CLDN18.2 are emerging as attractive tumor-associated antigens (TAAs) for the clinical development of CLDN-targeted therapies (Katoh and Katoh, 2024). For example, CLDN18.2 protein is overexpressed as a result of redistribution from the tight junction to non-junctional cell-surface membrane owing to dysregulated polarity in G/GEJ tumor cells (Nakayama et al., 2024).

CLDN18.2 (stomach isoform) is almost identical to CLDN18.1 (lung isoform) except for the N-terminal cytoplasmic region, the first transmembrane domain and ECL1 derived from isoform-specific first exons (Niimi et al., 2001; Katoh et al., 2024b). CLDN18.2 positivity in an experimental immunohistochemical (IHC) study, defined as 2+ (moderate) to 3+ (strong) staining in ≥60% of tumor cells (TCs) using rabbit antiserum against an epitope derived from CLDN18.2 ECL1, was detected in 56% (37/66) of gastric cancer cases, 60% (6/10) of pancreatic ductal adenocarcinoma (PDAC) cases, 50% (11/22) of esophageal adenocarcinoma cases and 10% (4/42) of ovarian adenocarcinoma cases (Sahin et al., 2008). Cut-off values for CLDN18.2 IHC in clinical settings are confusing, because different diagnostic antibodies and cut-offs are used in clinical trials for CLDN18.2 targeted therapeutics. For example, CLDN18.2 positivity in clinical IHC studies, defined as 2+/3+ membranous staining in ≥75% of TC using mouse 43-14A mAb against common CTR of CLDN18.2 and CLDN18.1 (≥2+ staining in ≥75% of TCs using 43-14A), was detected in 38.4% (1730/4507) ∼ 49.3% (256/519) of G/GEJ cancer patients (Murtazaalieva et al., 2026; Shitara et al., 2024; Stratton et al., 2025; Terán et al., 2026), although CLDN18.2 positivity with 43-14A diagnostic antibody in patients with lung, ovarian and pancreatic cancers remains to be further validated owing to its cross-reactivity to CLDN18.1.

The CLDN18.2-specific region in ECL1 has been applied as an epitope for the development of CLDN18.2-targeted therapies (Katoh and Katoh, 2024; Nakayama et al., 2024), which was expected to prevent cross-reaction to CLDN18.1 and reduce pulmonary adverse effects. Recent progress on CLDN18.2-directed therapeutic modalities (Figure 2), including mAbs, antibody–drug conjugates (ADCs), bispecific antibodies (bsAbs) and cell-based therapies, such as chimeric antigen receptor T (CAR-T) cells and T cells expressing a T-cell antigen coupler (TAC-T), will be reviewed in this section.

**FIGURE 2:**
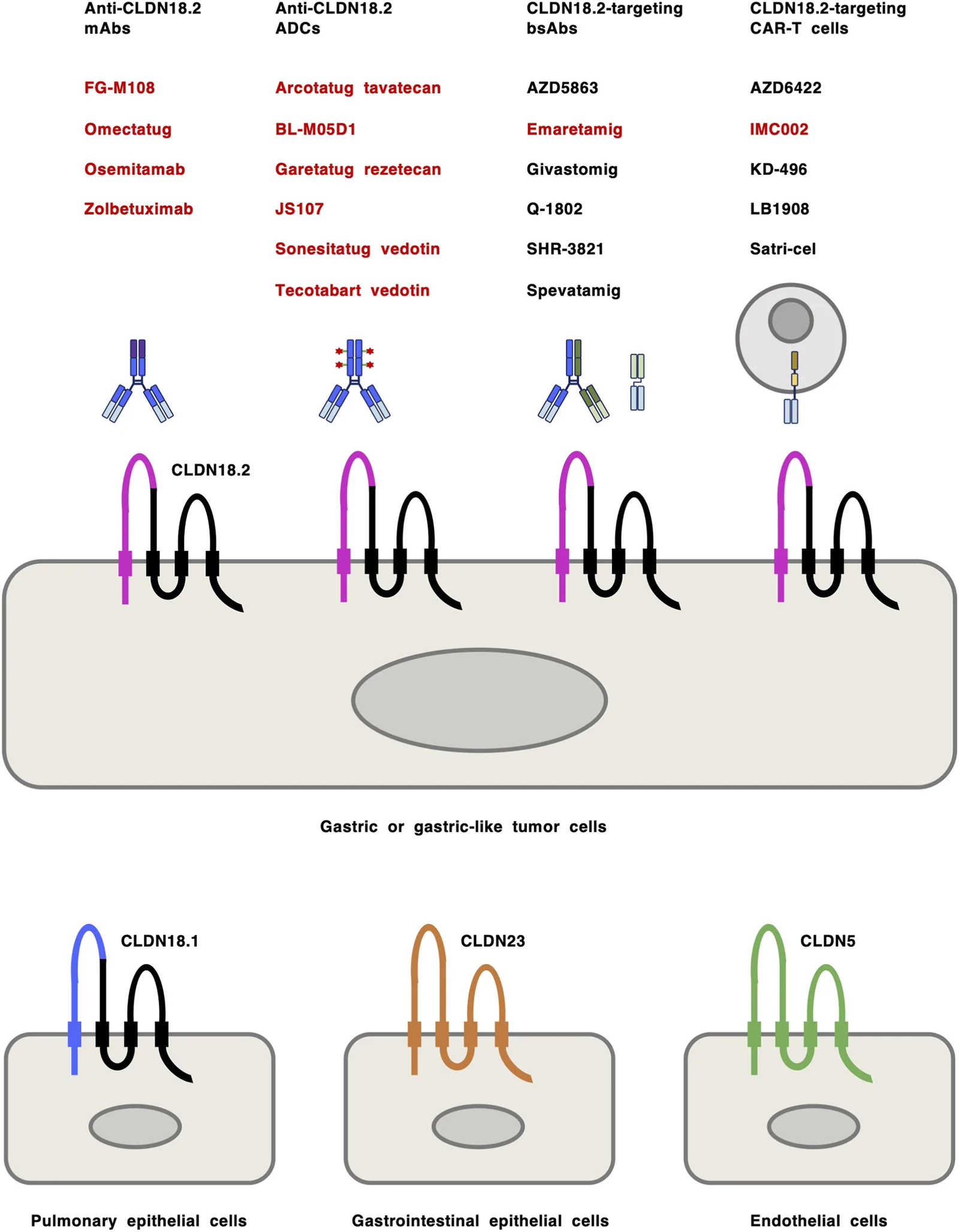
Claudin-18 isoform 2 (CLDN18.2)-targeted biologics. CLDN18.2 (pink continued on black) and CLDN18.1 (blue continued on black) are splicing variants derived from the *CLDN18* gene using alternative promoters. CLDN18.2 is overexpressed in approximately 40% of gastric and gastroesophageal junction cancers, whereas CLDN18.1, CLDN23 (orange) and CLDN5 (green) are expressed in pulmonary epithelial cells, gastrointestinal epithelial cells and endothelial cells, respectively. Anti-CLDN18.2 monoclonal antibodies (mAbs; FG-M108, omectatug, osemitamab and zolbetuximab), anti-CLDN18.2 antibody‒drug conjugates (ADCs; arcotatug tavatecan, BL-M05D1, garetatug rezetecan, JS107, sonesitatug vedotin and tecotabart vedotin), CLDN18.2-targeting bispecific antibodies (bsAbs; AZD5863, emaretamig, givastomig, Q-1802, SHR-3821 and spevatamig) and CLDN18.2-targeting chimeric antigen receptor T (CAR-T) cells (AZD6422, IMC002, KD-496, LB1908 and satricabtagene autoleucel [Satri-cel]) are in clinical development for patients with CLDN18.2-positive solid tumors. Single-agent objective response rates of mAb (omectatug), all ADCs mentioned above, bsAbs (emaretamig, givastomig, Q-1802 and SHR-3821) and CAR-T cells (IMC002, KD-496, LB1908 and Satri-cel) are higher than that of pioneering mAb, zolbetuximab. Gastrointestinal toxicity, nausea and vomiting are common on-target/off-tumor adverse effects of CLDN18.2-targeted biologics, although these adverse effects are mitigated for arcotatug tavatecan and givastomig owing to silencing of fragment crystallizable (Fc) region in their antibody format. Cytokine release syndrome is a specific adverse effect for Fc-efficient bispecific T-cell engagers, such as emaretamig, and CAR-T cells. Biologics in randomized controlled phase 3 clinical trials are shown in red, while those in early-phase clinical trials are shown in black. Zolbetuximab plus chemotherapy is approved for the first-line treatment of CLDN18.2-positive gastric and gastroesophageal junction (G/GEJ) cancer patients, while results of phase 3 clinical trials of other CLDN18.2-targeting biologics are anticipated with expectations for improved clinical activities and mitigated adverse events. In contrast, early-phase clinical trials of anti-CLDN18.2 therapeutics are ongoing to explore the possibility of indication expansion beyond G/GEJ cancers, such as pancreatic ductal adenocarcinoma, ovarian adenocarcinoma and non-small cell lung cancer.

### 2.1 Anti-CLDN18.2 mAbs

Representative anti-CLDN18.2 mAbs in clinical trials (Table 1) include FG-M108 (Liu F. et al., 2024), omectatug (ASKB589) (Zhang et al., 2023), osemitamab (TST001) (Janjigian et al., 2024) and zolbetuximab (Türeci et al., 2019). These therapeutic antibodies in the immunoglobulin G1 (IgG1) format induce fragment crystallizable (Fc) region-mediated antitumor effects, such as antibody-dependent cellular cytotoxicity and phagocytosis (ADCC and ADCP) and complement-dependent cytotoxicity (CDC) (Crescioli et al., 2025; Paul et al., 2024). Zolbetuximab is a first-generation chimeric mAb and consists of variable antigen-binding domains derived from mouse IgG1 and constant domains derived from human IgG1, whereas FG-M108, omectatug and osemitamab are second-generation human/humanized mAbs engineered for higher binding affinities and enhanced effector functions. In preclinical experiments, compared with zolbetuximab, FG-M108, with an afucosylated Fc region, achieved increased ADCC (Liu F. et al., 2024), and compared with the zolbetuximab analog, osemitamab enhanced ADCC, ADCP and CDC activities (Janjigian et al., 2024). Apart from having varying degrees of antitumor effects, anti-CLDN18.2 mAbs elicit common on-target/off-tumor adverse effects, such as gastrointestinal toxicity, nausea and vomiting.

**TABLE 1:** CLDN18.2-targeting monoclonal antibodies (mAbs) in clinical trials.

mAb	Format	Phase	Clinical trial ID	Design	Representative results	Status
FG-M108	Hs IgG1	1	NCT04894825	Mono	Single-agent ORR, N/A	Unknown
		3	NCT06177041	Combo-C vs. C	mOS and mPFS, N/A	Recruiting
Omectatug (ASKB589)	Hs IgG1	1/2	NCT04632108	Mono or combo	Single-agent ORR, 22%	Active NR
		3	NCT06206733	Combo-C/I vs. C/I	mOS and mPFS, N/A	Active NR
Osemitamab (TST001)	Hs IgG1	1/2	NCT04396821	Mono or combo	Single-agent ORR, N/A	Active NR
		3	NCT06093425	Combo-C vs. C Combo-C/I vs. C/I	mOS and mPFS, N/A	Not yet
Zolbetuximab (IMAB362)	Mm-Hs chimeric IgG1	2	NCT01197885	Mono	Single-agent ORR, 9%	Completed
		3	NCT03504397	Combo-C vs. C *	mOS, 18.23 vs. 15.54 months (HR 0.75, 95% CI 0.60–0.94, P = 0.005) mPFS, 10.61 vs. 8.67 months (HR 0.75, 95% CI 0.60–0.94, P = 0.0066)	Active NR
		3	NCT03653507	Combo-C vs. C **	mOS, 14.39 vs. 12.16 months (HR 0.771, 95% CI 0.615–0.965, P = 0.0118) mPFS, 8.21 vs. 6.80 months (HR 0.687, 95% CI 0.544–0.866, P = 0.0007)	Active NR
		3	NCT06901531	Combo-C/I vs. C/I	mOS and mPFS, N/A	Recruiting

Active NR, active, not recruiting; C, chemotherapy; CI, confidence interval; Combo, combination therapy; Hs, human or humanized; HR, hazard ratio; I, immune checkpoint inhibitor; Mm-Hs, mouse–human chimeric; Mono, monotherapy; mOS, median overall survival; mPFS, median progression-free survival; N/A, not available; Not yet, not yet recruiting; ORR, objective response rate. * SPOTLIGHT, study for the first-line treatment of CLDN18.2-positive G/GEJ, cancer patients, zolbetuximab plus modified folinic acid, fluorouracil, and oxaliplatin chemotherapy (mFOLFOX6) group (n = 283) vs. placebo plus mFOLFOX6 group (n = 282). ** GLOW, study for the first-line treatment of CLDN18.2-positive G/GEJ, cancer patients, zolbetuximab plus capecitabine and oxaliplatin chemotherapy (CAPOX) group (n = 254) vs. placebo plus mFOLFOX6 group (n = 253).

Zolbetuximab monotherapy resulted in an objective response rate (ORR) of 9% (4/43) and manageable safety profiles for CLDN18.2-positive (defined as ≥2+ staining in ≥50% of TCs using 43-14A) G/GEJ cancer patients in a phase 2 MONO study (NCT01197885) (Türeci et al., 2019). A combination strategy with conventional chemotherapies was then applied for zolbetuximab because of its limited single-agent ORR. Zolbetuximab plus standard chemotherapies revealed clinical benefits for first-line treatment of CLDN18.2-positive G/GEJ cancer patients (defined as ≥2+ staining in ≥75% of TCs using 43-14A) compared with placebo plus chemotherapies, such as the median overall survival (mOS) of 18.2/14.4 versus 15.5/12.2 months and median progression-free survival (mPFS) of 10.6/8.2 versus 8.7/6.8 months in registry phase three SPOTLIGHT study (NCT03504397) (Shitara et al., 2023) and GLOW study (NCT03653507) (Shah et al., 2023), respectively [modified folinic acid, fluorouracil, and oxaliplatin chemotherapy (mFOLFOX6) in the SPOTLIGHT study and capecitabine and oxaliplatin chemotherapy (CAPOX) in the GLOW study. Detailed statistics are presented in Table 1]. In addition, the use of zolbetuximab plus a chemotherapy and immune checkpoint inhibitor (ICI) for first-line treatment of CLDN18.2-positive G/GEJ cancer patients resulted in a combination ORR of 68% (32/47) and an mPFS of 14.8 months in a phase 2 ILUSTRO study (NCT03505320) (Shitara et al., 2026), which led to an ongoing phase three LUCERNA study (NCT06901531) to validate the clinical benefits of zolbetuximab-based chemoimmunotherapy. At present, zolbetuximab in combination with fluoropyrimidine- and platinum-containing chemotherapy is approved for the first-line treatment of CLDN18.2-positive G/GEJ cancer patients by the US FDA.

In contrast, omectatug achieved a single-agent ORR of 22% (2/9) in a phase 1/2 clinical trial (NCT04632108) (Zhang et al., 2023). Omectatug plus CAPOX and ICI achieved a combination ORR of 76% (35/46) and mPFS of 12.5 months in another phase 1/2 clinical trial (NCT05632939) (Shen et al., 2025), while FG-M108 plus CAPOX achieved a combination ORR of 78% (28/36) in a phase 1/2 clinical trial (NCT06177041) (Liu F. et al., 2024). Osemitamab plus CAPOX and ICI for first-line treatment of CLDN18.2-positive (defined as ≥2+ staining in ≥40% of TCs using 14G11 raised against CLDN18.2 ECL1) G/GEJ cancer patients revealed a combination ORR of 68%, mOS of 21.7 months and mPFS of 16.6 months in a phase 1/2 TranStar102 study (NCT04495296) (Alderson et al., 2024; Liu D et al., 2025). On the basis of these promising results, FG-M108, omectatug and osemitamab were subsequently used in phase three randomized controlled trials (NCT06177041, NCT06206733 and NCT06093425, respectively) to confirm clinical relevance of these ADCs versus placebo in combination with chemotherapy or chemoimmunotherapy for the first-line treatment of CLDN18.2-positive G/GEJ cancer patients (Table 1).

Differences in the cut-off value of CLDN18.2 positivity in osemitamab trials (40%) versus zolbetuximab trials (75%), reflecting the specificities of diagnostic antibodies (14G11 versus 43-14A) or formats of therapeutic antibodies (engineered humanized IgG1 versus mouse-human chimeric IgG1), would contribute to the expansion of eligible patients for CNDN18.2-targeted therapies. Comparative studies of the effects of second-generation anti-CLDN18.2 mAbs and zolbetuximab on mOS and mPFS are anticipated for the optimal management of G/GEJ cancer patients in the future.

### 2.2 CLDN18.2-targeting ADCs

ADCs are mAb derivatives that are linked to cytotoxic payloads, such as monomethyl auristatin E (MMAE) and topoisomerase I inhibitor (TOP1i), using cleavable or non-cleavable chemical linkers. ADCs targeting transmembrane or membrane-anchored proteins exert antitumor activity on the basis of (i) direct effects on TAA-positive cancer cells via binding, internalization and cytoplasmic release of toxic payloads (TAA-targeted drug delivery), (ii) indirect effects on TAA-negative cancer cells via payload release from TAA-positive cancer cells (bystander effects), and (iii) effects on TAA-positive cancer cells via ADCC, ADCP and CDC (Fc-mediated effector functions) (Drago et al., 2021; Fu et al., 2022), although Fc-mediated immune responses are enhanced or silenced by Fc engineering to address the trade-off between antitumor effects and adverse effects (Crescioli et al., 2025). ADCs targeting BCMA, CD19, CD22 and CD30 have been approved for the treatment of patients with hematological malignancies, while ADCs targeting HER2, MET, NECTIN4 and TROP2 have been approved for the treatment of patients with solid tumors (Dumontet et al., 2023; Katoh et al., 2025).

Representative CLDN18.2-targeting ADCs in clinical trials (Table 2) include arcotatug tavatecan (IBI343 or TAK-921) (Liu J. et al., 2025), ASP546C (XNW27011) (Yu et al., 2025), ATG-022 (Ma et al., 2025), BL-M05D1 (Cheng et al., 2026), ciletatug vedotin (RC118) (Liu T. et al., 2024), garetatug rezetecan (SHR-A1904) (Ruan et al., 2025b), JS107 (Xu et al., 2025a), sonesitatug vedotin (AZD0901 or CMG901) (Ruan et al., 2025a) and tecotabart vedotin (LM-302) (Bai et al., 2025). Payloads of ATG-022, ciletatug vedotin, JS107, sonesitatug vedotin and tecotabart vedotin are MMAEs, and those of others are TOP1i (Katoh et al., 2025). In contrast, the drug–antibody ratios (DARs) of arcotatug tavatecan, garetatug rezetecan, sonesitatug vedotin and tecotabart vedotin are 4, and that of ASP546C is 8 (Conilh et al., 2026). Most of these ADCs are designed to enhance antitumor activity via payload delivery, bystander effects and Fc-mediated effector functions, whereas arcotatug tavatecan with Fc silencing is designed to attenuate ADCC and CDC to mitigate on-target/off-tumor gastrointestinal toxicity (Liu J. et al., 2025).

**TABLE 2:** CLDN18.2-targeting antibody–drug conjugates (ADCs) in clinical trials.

ADC	Payload (DAR)	Phase	Clinical trial ID	Design	Results	Status
Arcotatug tavatecan (IBI343)	TOP1i (4)	1	NCT05458219	Mono	Single-agent ORR, 35%	Recruiting
		3	NCT06238843	Mono vs. C	mOS and mPFS, N/A	Recruiting
ASP546C (XNW27011)	TOP1i (8)	1/2	CTR20231735	Mono	Single-agent ORR, 39%	Recruiting
ATG-022	MMAE (N/A)	1/2	NCT05718895	Mono	Single-agent ORR, 42%	Recruiting
BL-M05D1	TOP1i (N/A)	1	NCT06349811	Mono	Single-agent ORR, 35%	Recruiting
		3	NCT07518147	Mono vs. C	mOS and mPFS, N/A	Recruiting
Ciletatug vedotin (RC118)	MMAE (N/A)	1/2	NCT05205850	Mono	Single-agent ORR, 47%	Recruiting
Garetatug rezetecan (SHR-A1904)	TOP1i (4)	1	NCT04877717	Mono	Single-agent ORR, 23%	Active NR
		3	NCT06649292	Mono vs. C	mOS and mPFS, N/A	Recruiting
JS107	MMAE (N/A)	1	NCT05502393	Mono	Single-agent ORR, 35%	Unknown
		3	NCT07284134	Mono vs. C	mOS and mPFS, N/A	Recruiting
Sonesitatug vedotin (AZD0901 or CMG901)	MMAE (4)	1	NCT04805307	Mono	Single-agent ORR, 33%	Completed
		3	NCT06346392	Mono vs. C	mOS and mPFS, N/A	Recruiting
Tecotabart vedotin (LM-302)	MMAE (4)	1/2	NCT05161390	Mono	Single-agent ORR, 33%	Active NR
		3	NCT06351020	Mono vs. C	mOS and mPFS, N/A	Recruiting

Active NR, active, not recruiting; C, chemotherapy; DAR, drug–antibody ratio; MMAE, monomethyl auristatin E; mono, monotherapy; mOS, median overall survival; mPFS, median progression-free survival; N/A, not available; ORR, objective response rate; TOP1i, topoisomerase I inhibitor.

Early-phase clinical trials revealed manageable safety profiles and promising antitumor effects of representative CLDN18.2-targeting ADCs (Table 2). Single-agent ORRs were 35% (17/48) for arcotatug tavatecan (Liu J. et al., 2025), 39% (29/75) for ASP546C (Yu et al., 2025), 35% (28/79) for BL-M05D1 (Cheng et al., 2026), 47% (8/17) for ciletatug vedotin (Liu T. et al., 2024), 23% (17/74) for garetatug rezetecan (Ruan et al., 2025b), 35% (8/23) for JS107 (Xu et al., 2025a), 33% (31/93) for sonesitatug vedotin (Ruan et al., 2025a) and 33% (17/52) for tecotabart vedotin (Bai et al., 2025) in G/GEJ cancer patients with trial-specific definition of CLDN18.2 positivity: for example, arcotatug tavatecan, ≥2+ staining in ≥75% of TCs using 43-14A; garetatug rezetecan, ≥2+ staining in ≥50% of TCs using CLDN18.2-specific EPR19202; and sonesitatug vedotin, ≥2+ staining in ≥20% of TCs using MEDx. In contrast, the mOS and mPFS for sonesitatug vedotin were 11.8 and 4.8 months, respectively (Ruan et al., 2025a), and those for tecotabart vedotin were 10.9 and 4.9 months, respectively (Bai et al., 2025). In addition to promising antitumor effects, manageable safety profiles were also revealed: common adverse events of anti-CLDN18.2 ADCs included hematological and gastrointestinal toxicity.

Phase three randomized controlled clinical trials of arcotatug tavatecan (NCT06238843, G-HOPE-001 study), BL-M05D1 (NCT07518147), garetatug rezetecan (NCT06649292), JS107 (NCT07284134), sonesitatug vedotin (NCT06346392, CLARITY-Gastric 01 study) and tecotabart vedotin (NCT06351020) are ongoing to compare the efficacy and safety of ADC monotherapy versus the investigator’s choice of standard therapy (docetaxel, irinotecan, paclitaxel or others) for the second-line or later treatment of CLDN18.2-positive G/GEJ cancer patients (Table 2).

Engineered human/humanized anti-CLDN18.2 ADCs that have substantially evolved from a mouse-human chimeric anti-CLDN18.2 mA b might be appropriate modalities for CLDN18.2-targeted therapy. Future comparative studies on combination chemotherapy with zolbetuximab versus monotherapies of anti-CLDN18.2 ADCs, especially arcotatug tavatecan monotherapy, in the first-line setting of real-world clinical practice are anticipated from viewpoints of cost-effectiveness and quality of life.

### 2.3 CLDN18.2-targeting bsAbs

bsAbs are mAb derivatives that target dual antigens/epitopes in diverse formats, such as multivalent antigen/epitope-binding domains (1 + 1, 1 + 2 or 2 + 2 for dual targets) and Fc-engineering (enhanced, wild type or silenced). bsAbs are functionally classified into (i) CD3-dependent T-cell engagers bridging TAAs on cancer cells and CD3 antigens on T cells (TAA x CD3); (ii) 4-1BB-dependent T-cell engagers (TAA x 4-1BB); (iii) dual TAA binders (for example, TAA x PD-L1, TAA x CD47 and EGFR x MET); and (iv) dual ICIs (for example, PD-1 x CTLA4 and PD-L1 x CTLA4) (Fontana et al., 2026; Goebeler et al., 2024; Klein et al., 2024).

Representative CLDN18.2-targeting bsAbs in clinical trials (Table 3) include AZD5863 (Gaspar et al., 2025), emaretamig (ASP2138) (Shitara et al., 2025a), givastomig (ABL111 or TJ033721) (Gao et al., 2023; Ku et al., 2025), IBI389 (Li X. et al., 2026), PM1032 (Guo et al., 2024), Q-1802 (Wang et al., 2023b), QLS31905 (Wang et al., 2023a), SHR-3821 (Gou et al., 2026) and spevatamig (PT886) (Li et al., 2022; Saeed et al., 2026). AZD5863, emaretamig, IBI389 and QLS31905 are CD3-dependent T-cell engagers (CLDN18.2 x CD3) that activate T-cell receptor (TCR) signaling in tumor-infiltrating T cells (TILs) and redirect cytotoxic T cells toward CLDN18.2-positive cancer cells, while givastomig, PM1032 and SHR-3821 are 4-1BB-dependent T-cell engagers (CLDN18.2 x 4-1BB) that activate costimulatory 4-1BB signaling in TILs of CLDN18.2-positive tumors and induce antitumor cytotoxicity. In contrast, Q-1802 and spevatamig are dual TAA binders: Q-1802 (CLDN18.2 x PD-L1) blocks coinhibitory PD-L1/PD-1 signaling in TILs and enhances antitumor immunity, and spevatamig (CLDN18.2 x CD47) inhibits “don’t eat me” signaling in tumor-associated macrophages (TAMs) and promotes the phagocytosis of CLDN18.2-positive cancer cells (Katoh and Katoh, 2024).

**TABLE 3:** CLDN18.2-targeting bispecific antibodies (bsAbs) in clinical trials.

bsAb	Targets	Phase	Clinical trial ID	Design	Representative results	Status
AZD5863	CLDN18.2 x CD3	1/2	NCT06005493	Mono	Single-agent ORR, N/A	Recruiting
Emaretamig (ASP2138)	CLDN18.2 x CD3	1	NCT05365581	Mono or combo	Single-agent ORR, 14%	Recruiting
		3	NCT07673887	Combo-C/I vs. C/I	mOS and mPFS, N/A	Recruiting
Givastomig (ABL111 or TJ033721)	CLDN18.2 x 4-1BB	1	NCT04900818	Mono or combo	Single-agent ORR, 16%	Recruiting
		2	NCT07432295	Combo-C/I vs. C/I	mOS and mPFS, N/A	Recruiting
IBI389	CLDN18.2 x CD3	1	NCT05164458	Mono or Combo-I	Single-agent ORR, 22%	Unknown
PM1032	CLDN18.2 x 4-1BB	1/2	NCT05839106	Mono	Single-agent ORR, 20%	Unknown
Q-1802	CLDN18.2 x PD-L1	1	NCT04856150	Mono	Single-agent ORR, 22%	Unknown
QLS31905	CLDN18.2 × CD3	1	NCT05278832	Mono	Single-agent ORR, 11%	Unknown
SHR-3821	CLDN18.2 x 4-1BB	1	NCT06618651	Mono	Single-agent ORR, 29%	Recruiting
Spevatamig (PT886)	CLDN18.2 × CD47	1/2	NCT05482893	Mono or combo	Single-agent ORR, N/A	Recruiting

C, chemotherapy; Combo, combination therapy; I, immune checkpoint inhibitor; Mono, monotherapy; mOS, median overall survival; mPFS, median progression-free survival; N/A, not available; Not yet, not yet recruiting; ORR, objective response rate.

Bispecific T-cell engagers induce functional activation of TILs, including the release of perforin and granzyme for direct killing of cancer cells and the production of interferon-γ (IFN-γ) and tumor necrosis factor-α (TNF-α) for subsequent activation of myeloid and endothelial cells. Because systemic elevation of interleukin-6 (IL-6) and other inflammatory cytokines derived from activated immune cells leads to cytokine release syndrome (CRS) (Radtke et al., 2025), Fc-mediated effector functions are removed from bispecific T-cell engagers, such as AZD5863 with Fc mutations (L234A, L235A and G237A) and givastomig with Fc mutation (N300A), to mitigate immune-related adverse effects (Gaspar et al., 2025; Gao et al., 2023).

Givastomig with Fc silencing achieved a single-agent ORR of 16% (7/43) but produced no symptoms of systemic immune response in CLDN18.2-positive (defined as ≥1+ staining in ≥1% of TCs using CLDN18.2-specific SP455) G/GEJ cancer patients in a phase 1 clinical trial (NCT04900818) (Ku et al., 2025). In contrast, emaretamig and IBI389 revealed single-agent ORRs of 14% (5/36) and 22% (6/27) but also CRS in 43% and 60% of patients in phase 1 clinical trials NCT05365581 (Shitara et al., 2025a) and NCT05164458 (Li C et al., 2026), respectively. Because CRS can be dose-limiting and life-threatening, the clinical benefits of Fc-silenced T-cell engagers, such as AZD5863 and givastomig, are promising. Therefore, further evaluation of the antitumor effects and safety profiles of Fc-silenced T-cell engagers in advanced-stage clinical trials is anticipated.

Anti-CLDN18.2 bsAbs, except emaretamig, remain in relatively early phases of clinical trials (Table 3) than anti-CLDN18.2 mAbs (Table 1) and anti-CLDN18.2 ADCs (Table 2). Considering the striking ORRs of CLDN18.2-targeting ADCs (Table 2), the clinical development of CLDN18.2-targeting bsAbs might have a difficult road ahead from the perspective of business decisions.

### 2.4 CLDN18.2-targeting CAR-T and TAC-T cells

Conventional CAR-T cells are engineered autologous T cells that typically express CAR constructs with mAb-derived single-chain variable fragment (scFv) domains, CD8α/CD28-derived hinge and transmembrane domains, and 4-BB/CD28-derived costimulatory domains and CD3ζ-derived signaling domains (Cappell and Kochenderfer, 2023). As a result of scFv-mediated TAA-binding, CAR-T cells undergo clustering of CAR constructs and subsequent activation of downstream signaling, which leads to (i) perforin/granzyme-mediated direct killing of cancer cells, (ii) IFN-γ/TNF-α-mediated activation of innate immune cells for the production of inflammatory cytokines, and (iii) proliferation and persistence of CAR-T cells themselves in the tumor microenvironment (TME) (Larson and Maus, 2021). CAR-T cells targeting CD19 and B-cell maturation antigen (BCMA or TNFRSF17) are approved for the treatment of B-cell lymphoma/leukemia patients and multiple myeloma patients, respectively (Cappell and Kochenderfer, 2023), while investigational CAR-T cells targeting CLDN18.2, DLL3, EGFR and glypican 3 (GPC3) are in clinical trials for the treatment of solid tumor patients (Katoh and Katoh, 2024; Li et al., 2025). In addition, armed CAR-T cells (Prasad et al., 2026), TAC-T cells (Minguet et al., 2025) and allogeneic CAR-γ/δT cells (Zugasti et al., 2025) have been designed as next-generation engineered T cells to further improve clinical benefits of CAR-T therapy.

Representative CLDN18.2-targeting CAR-T and TAC-T cells in clinical trials (Table 4) include AZD6422 (Barrett et al., 2024), IMC002 (Luo et al., 2024), KD-496 (Xu et al., 2022), LB 1908 (Zhen et al., 2026), satricabtagene autoleucel (satri-cel or CT041) (Qi et al., 2024) and TAC01 (Morey et al., 2025). LB1908 and satri-cel are conventional CAR-T cells. AZD6422, armed with dominant-negative transforming growth factor β receptor II (dnTGFBR2); IMC002, engineered with the variable domain of the heavy chain of heavy chain antibody (VHH); and KD-496, dually targeting CLDN18.2 and NKG2D ligands (NKG2DL), are next-generation CAR-T cells (Katoh and Katoh, 2024). In contrast, compared with conventional CAR-T cells, TAC01 cells are engineered with a construct consisting of anti-CLDN18.2 scFv, anti-CD3 ScFv and CD4-derived transmembrane and cytoplasmic domains. These TAC-T cells demonstrate enhanced antitumor efficacy and increased tumor-infiltration potential (Morey et al., 2025).

**TABLE 4:** CLDN18.2-targeting cell therapies in clinical trials.

Cell therapy	Type	Target	Phase	Clinical trial ID	Design	Representative results	Status
AZD6422	CAR-T	CLDN18.2	1	NCT05981235	Mono	Single-agent ORR, N/A	Completed
IMC002	CAR-T	CLDN18.2	1	NCT05946226	Mono	Single-agent ORR, 67%	Unknown
			3	NCT07103668	Mono vs. C	mOS and mPFS, N/A	Recruiting
KD-496	CAR-T	CLDN18.2 x NKG2DL	1	NCT06134960	Mono	Single-agent ORR, 75%	Recruiting
LB1908	CAR-T	CLDN18.2	1	NCT05539430	Mono	Single-agent ORR, 30%	Active NR
Satri-cel (CT041)	CAR-T	CLDN18.2	1	NCT03874897	Mono or combo	Single-agent ORR, 55%	Completed
			1/2	NCT04581473	Mono vs. TPC	mPFS, 3.25 vs. 1.77 months (p < 0.0001)	Active NR
TAC01-CLDN18.2	TAC-T	CLDN18.2	1/2	NCT05862324	Mono	Single-agent ORR, N/A	Active NR

Active NR, active, not recruiting; C, chemotherapy; CAR-T, chimeric antigen receptor T cells; Combo, combination therapy; I, immune checkpoint inhibitor; Mono, monotherapy; mOS, median overall survival; mPFS, median progression-free survival; N/A, not available; Not yet, not yet recruiting; ORR, objective response rate; TAC-T, T cells expressing T-Cell Antigen Coupler; TPC, treatment of physician’s choice (paclitaxel, irinotecan or apatinib or anti-PD-1, antibody).

Conventional CAR-T cells, namely, LB1908 and satri-cel, achieved single-agent ORRs of 30% (3/10) and 55% (28/51) in CLDN18.2-positive (defined as ≥1+ staining in ≥50% of TCs and ≥2+ staining in ≥40% of TCs) G/GEJ cancer patients in NCT05539430 and NCT03874897 phase 1 clinical trials, respectively (Zhen et al., 2026; Qi et al., 2024). In a randomized phase 1/2 clinical trial (NCT04581473) (Qi et al., 2025a), satri-cel versus investigator’s choice of therapy for the treatment of CLDN18.2-positive G/GEJ cancer patients resulted in an mPFS of 3.25 months versus 1.77 months [hazard ratio (HR) 0.37, 95% confidence interval (CI) 0.24–0.56, one-sided log-rank p < 0.0001]. Common adverse events of LB1908 and satri-cel were hematological toxicities owing to preconditioning combination chemotherapy and on-tumor/off-tumor adverse effects, including gastrointestinal toxicity and CRS, but not immune effector cell-associated neurotoxicity syndrome (ICANS). Hematological toxicity was inevitable because of preconditioning combination chemotherapy, whereas CRS was manageable using anti-IL-6R mAb and systemic corticosteroids. These results of early-phase clinical trials demonstrated antitumor effects and manageable safety profiles of conventional CAR-T cells, especially satri-cel, for late-line treatment of G/GEJ cancer patients.

In contrast, next-generation CAR-T cells demonstrated strong single-agent activity. Specifically, IMC002 and KD-496 achieved single-agent ORRs of 67% (10/15) and 75% (3/4) in CLDN18.2-positive G/GEJ cancer patients and NKG2DL/CLDN18.2-double-positive gastric cancer patients in phase 1 clinical trials NCT05946226 and NCT06134960, respectively (Li X. et al., 2026; Qi et al., 2025b), while the adverse effects of IMC002 and KD-496 were similar to those of LB1908 and satri-cel. Because a complete response (CR) was achieved in 11% (2/18) of patients treated with IMC002, a phase three randomized controlled study of IMC002 versus investigator’s choice of therapy for third-line or later treatment of CLDN18.2-positive G/GEJ cancer patients (NCT07103668) is ongoing.

CLDN18.2-targeting CAR-T-cell therapies, such as IMC002 and satri-cel, are opening up a new therapeutic avenue for solid tumor patients; however, resistance mechanisms to CAR-T cells (immunosuppressive TME, heterogenous TAA expression and antigen loss) (Lamplugh et al., 2026) and high costs of autologous CAR-T cells (Depil et al., 2020) remain as crucial issues in this field. Therefore, clinical development of allogeneic armed CAR-T cells is anticipated to further improve the clinical benefits of CLDN18.2-targeted cell therapies in the future (Figure 1).

## 3 FGFR2b-targeted therapies

FGFR2b and FGFR2c consist of extracellular immunoglobulin-like (Ig-like) domains, single transmembrane domain and a cytoplasmic tyrosine kinase domain, which are generated from the *FGFR2* gene using mutually exclusive exons 8 versus 9 corresponding to the latter half of the third Ig-like domain (Katoh et al., 2024a). Epithelial-specific RNA-binding proteins (ESRP1 and ESRP2) induce exon 8 splicing into the FGFR2b isoform, and the repression of ESRPs in mesenchymal cells leads to exon 9 splicing into the FGFR2c isoform (Warzecha et al., 2009). FGFR2b receptors on epithelial cells transduce signals of FGF7, FGF10 and FGF22, whereas FGFR2c receptors on mesenchymal cells transduce signals of FGF2 (basic FGF or bFGF), FGF4, FGF6 and FGF9 (Ornitz and Itoh, 2015). Both epithelial FGFR2b and mesenchymal FGFR2c are expressed in gastric cancer (Yashiro et al., 2021) owing to epithelial–mesenchymal plasticity that is involved in malignant phenotypes, especially dormancy, drug resistance and recurrence (Katoh and Katoh, 2022; Zhang et al., 2025).

The *FGFR2* gene at human chromosome 10q26.13 is an oncogene that is overexpressed and activated in 16.6% (1310/7910) of G/GEJ cancers (Maron et al., 2026) owing to gene amplifications (chromosomal and extrachromosomal) and C-terminal fusions/rearrangements (Hung et al., 2022; Zingg et al., 2022). Clinical activities of FGFR inhibitors targeting both FGFR2b and FGFR2c isoforms, such as pan-FGFR inhibitors (erdafitinib and futibatinib), an FGFR1/2/3 inhibitor (pemigatinib) and a selective FGFR2 inhibitor (lirafugratinib), are relatively limited for gastric cancer patients with *FGFR2* amplifications in comparison with cholangiocarcinoma patients with *FGFR2* fusions/rearrangements (Katoh et al., 2024a; Schram et al., 2023). In contrast, an anti-FGFR2b mAb (bemarituzumab also known as FPA144) has been shown to have clinical activity and manageable safety profiles in early-stage clinical trials, and has been evaluated in phase three clinical trials for the treatment of G/GEJ cancer patients with FGFR2b overexpression (Wainberg et al., 2022). Recent progress on FGFR2b-directed mAbs, ADCs and bsAbs (Figure 3) will be reviewed in this section.

**FIGURE 3:**
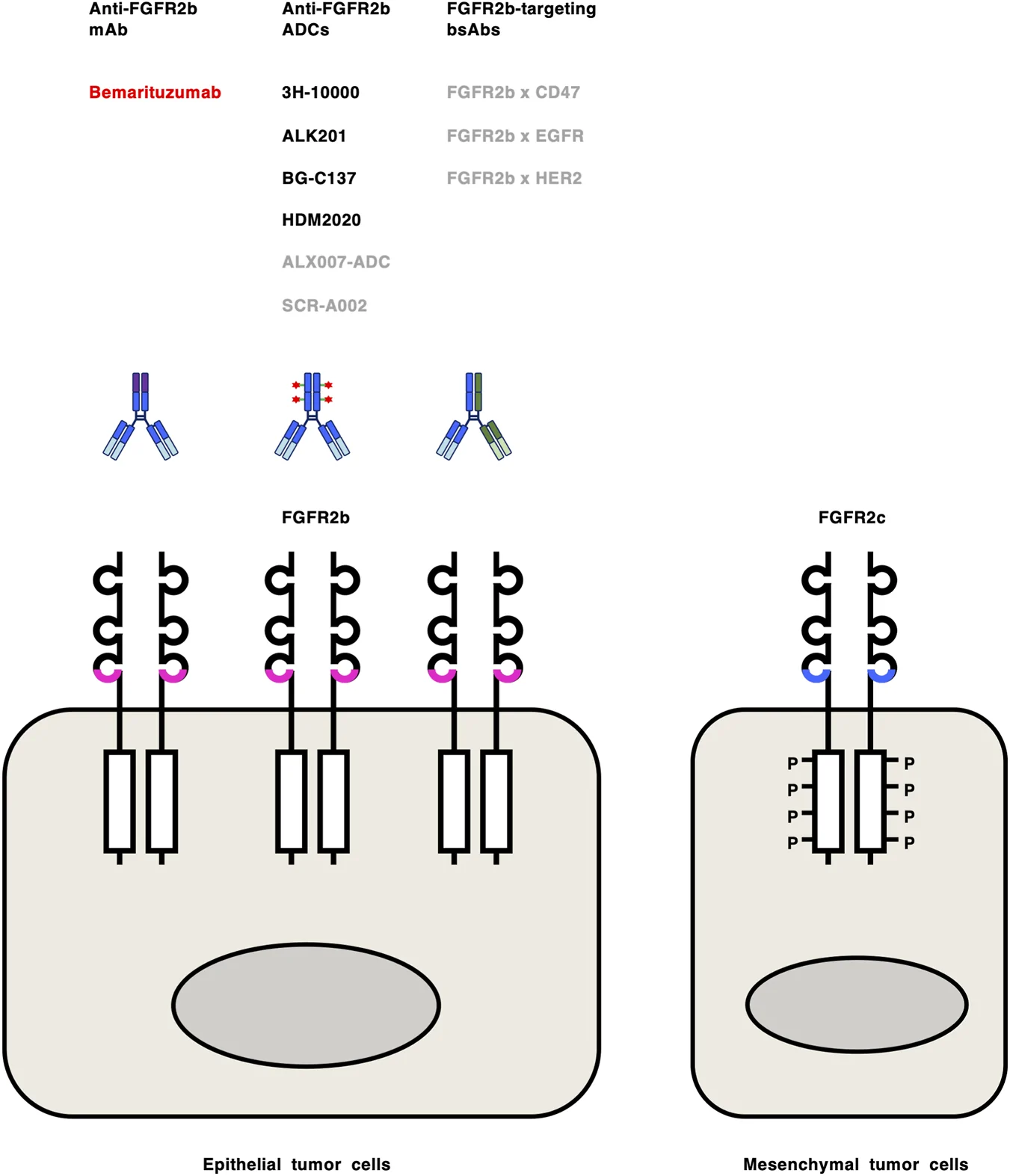
Fibroblast growth factor receptor 2 isoform b (FGFR2b)-targeted biologics. FGFR2b (pink in the third immunoglobulin [Ig]-like domain) and FGFR2c (blue in the third Ig-like domain) are splicing variants derived from the *FGFR2* gene that use alternative exclusive exons. Epithelial FGFR2b is overexpressed in approximately 20% of gastric and gastroesophageal junction cancers owing to gene amplifications, fusions or rearrangements. In contrast, mesenchymal FGFR2c is also expressed in gastric cancer as a result of epithelial–mesenchymal plasticity, which drives dormancy, drug resistance and recurrence. Anti-FGFR2b monoclonal antibody (mAb; bemarituzumab), anti-FGFR2b antibody‒drug conjugates (ADCs; 3H-10000, ALK201, BG-C137, HDM 2020, ALX007-ADC and SCR-A002) and FGFR2b-targeting bispecific antibodies (bsAbs; FGFR2b x CD47, FGFR2b x EGFR and FGFR2b x HER2) are investigational biologics for the treatment of patients with FGFR2b-positive solid tumors. Bemarituzumab in randomized, controlled phase 3 clinical trials is shown in red, while biologics in early-phase clinical trials and preclinical studies are shown in black and gray, respectively.

### 3.1 Bemarituzumab

Bemarituzumab is a humanized anti-FGFR2b mAb with an afucosylated IgG1 format. In preclinical experiments, bemarituzumab exhibited antitumor effects on FGFR2b-positive cancer cells via (i) inhibition of FGF7-dependent FGFR2b signaling and proliferation and (ii) enhancement of peripheral blood mononuclear cell (PBMC)-mediated ADCC but also elicited dose-dependent reversible corneal atrophy in rats and cynomolgus monkeys (Xiang et al., 2021).

A phase 1 clinical trial of bemarituzumab monotherapy (FPA144-001 study, NCT02318329) revealed an ORR of 16% (5/32) in FGFR2b-positive (defined as ≥2+ staining in ≥10% of TCs using FGFR2b-specific FPR2-D) G/GEJ cancer patients and adverse effects, such as gastrointestinal, hematological and ophthalmological toxicity (Catenacci et al., 2020). The single-agent ORR of bemarituzumab, comparable to that of the ICI pembrolizumab (15.5%, 23/148) (Fuchs et al., 2018), was promising.

A phase 2 randomized controlled trial of bemarituzumab versus placebo in combination with mFOLFOX6 chemotherapy (FIGHT study, NCT03694522) revealed favorable clinical activities: combination ORRs, 48% versus 33%; mOS, 19.2 versus 13.5 months (HR 0.77, 95% CI 0.52–1.14); and mPFS, 9.5 versus 7.4 months (HR 0.72, 95% CI 0.49–1.08). In contrast, adverse events leading to treatment discontinuation occurred in 41% (31/76) of the bemarituzumab group versus 5% (4/77) of the placebo group, among which ophthalmological toxicities, such as dry eye, keratitis and corneal epithelial defect, occurred in 32% of the bemarituzumab group but not in the placebo group (Wainberg et al., 2022).

Phase three randomized controlled trials of bemarituzumab versus placebo in combination with chemotherapy (FORTITUDE-101 study, NCT05052801) or chemoimmunotherapy (FORTITUDE-102 study, NCT05111626) for the first-line treatment of FGFR2b-positive (defined as ≥2+ staining in ≥10% of TCs) G/GEJ cancer patients began in 2022. Interim analysis on the FORTITUDE-101 study demonstrated significant survival benefits [mOS, 17.9 versus 12.5 months (HR 0.61, 95% CI 0.43–0.86, P = 0.005); and mPFS, 8.6 versus 6.7 months (HR 0.71, 95% CI 0.53–0.95, P = 0.019)] and also higher incidence of grade ≥3 treatment-emergent adverse events [60.0% (165/275) versus 18.4% (49/267), mainly owing to ocular toxicities]; however, descriptive analysis after longer follow-up revealed an attenuated clinical benefit [mOS, 14.5 versus 13.2 months (HR 0.82, 95% CI 0.62–1.08)] (Rha et al., 2025). The FORTITUDE-101 study was completed in April 2026, whereas the FORTITUDE-102 study was terminated owing to business decision of AMGEN (Table 5). Clinical activities and safety profiles of bemarituzumab-based combination therapy in the FORTITUDE-101 study with subset analyses on biomarkers and ethnicity are anticipated but not yet available as of 26 August 2026.

**TABLE 5:** FGFR2b-targeting biologics in clinical trials and preclinical studies.

Biologics	Format	Phase	Clinical trial ID	Design	Representative results	Status
Bemarituzumab	mAb	1	NCT02318329	Mono	Single-agent ORR, 16%	Completed
		2	NCT03694522	Combo-C vs. C	Combo ORR, 48% vs. 33%; mOS, 19.2 vs. 13.5 months (HR 0.77, 95% CI 0.52–1.14); mPFS, 9.5 vs. 7.4 months (HR 0.72, 95% CI 0.49–1.08)	Completed
		3	NCT05052801	Combo-C vs. C	mOS, 17.9 vs. 12.5 months (HR 0.61, 95% CI 0.43–0.86, P = 0.005) mPFS, 8.6 vs. 6.7 months (HR 0.71, 95% CI 0.53–0.95, P = 0.019)	Completed
		3	NCT05111626	Combo-C/I vs. C/I	mOS and mPFS, N/A	Terminated
3H-10000	ADC, MMAE	1/2	NCT07354711	Mono	Single-agent ORR, N/A	Recruiting
ALK201	ADC, TOP1i	1/2	NCT06656390	Mono	Single-agent ORR, 24%	Recruiting
BG-C137	ADC, TOP1i	1	NCT06625593	Mono	Single-agent ORR, N/A	Recruiting
HDM2020	ADC, TOP1i	1	NCT07098052	Mono	Single-agent ORR, N/A	Recruiting
ALX007-ADC	ADC, TOP1i	Pre	​	​	​	​
SCR-A002	ADC, TOP1i	Pre	​	​	​	​
Unnamed	bsAb, FGFR2b x CD47	Pre	​	​	​	​
Unnamed	bsAb, FGFR2b x EGFR	Pre	​	​	​	​
Unnamed	bsAb, FGFR2b x HER2	Pre	​	​	​	​

Active NR, active, not recruiting; ADC, antibody–drug conjugate; C, chemotherapy; Combo, combination therapy; HR, hazard ratio; I, immune checkpoint inhibitor; mAb, monoclonal antibody; MMAE, monomethyl auristatin E; mono, monotherapy; mOS, median overall survival; mPFS, median progression-free survival; N/A, not available; Not yet, not yet recruiting; ORR, objective response rate; Pre, preclinical; TOP1i, topoisomerase I inhibitor.

### 3.2 Next-generation FGFR2b-targeted biologics

On the basis of their promising clinical activities and concerns over the ophthalmological toxicities of bemarituzumab as well as a paradigm shift in biologics from mAb to ADC and bsAb platforms, investigational anti-FGFR2b mAb derivatives have been designed for clinical development (Table 5): 3H-10000 (Gao et al., 2026) is an anti-FGFR2b human ADC with MMAE payloads; ALK201 (Pan et al., 2025), ALX007-ADC (An et al., 2026), BG-C137 (Xu Y. et al., 2025), HDM 2020 (Xu R. H. et al., 2025b) and SCR-A002 (Fu et al., 2024) are anti-FGFR2b ADCs with TOP1i payloads; and several unnamed bsAbs are dual TAA binders (FGFR2b x CD47, FGFR2b x EGFR and FGFR2b x HER2) (Wienclaw and Kipp, 2025; Zhu et al., 2026).

In preclinical studies, compared with bemarituzumab, 3H-10000 exerted antitumor effects through FGFR2b-targeted MMAE delivery, bystander effects and Fc-mediated cytotoxic effects and mitigated corneal toxicity (Gao et al., 2026); ALK201 exhibited antitumor effects through direct payload delivery, bystander effects and ADCC, even in bemarituzumab-resistant patient-derived xenograft (PDX) models, but reversed corneal atrophy in cynomolgus monkeys in a dose-dependent manner (Pan et al., 2025); ALX007-naked mAb and ALX007-ADC resulted in milder corneal dystrophy than did bemarituzumab owing to the epitope design for intact FGF7/10-FGFR2b signaling (An et al., 2026); BG-C137 ADC spared corneal dystrophy in cynomolgus monkeys owing to weaker FGFR2b signaling inhibition (Xu Y. et al., 2025); and FGFR2b x EGFR bsAbs functioned as partial FGFR2b blocker that weakly inhibited FGF7 signaling but spared FGF10 signaling (Zhu et al., 2026). Notably, next-generation FGFR2b-targeting biologics have been designed to mitigate on-tumor/off-tumor adverse effects of bemarituzumab, especially ophthalmological toxicity.

3H-10000 and ALK201 proceeded to phase 1/2 clinical trials NCT07354711 and NCT06656390, respectively, while BG-C137 and HDM2020 entered phase 1 clinical trials NCT06625593 and NCT07098052, respectively. Because ALK201 achieved a single-agent ORR of 24% (6/25) and had manageable safety profiles, including nausea (45%), anemia (21%), vomiting (21%) and grade 1 ocular adverse effects (11%) in the NCT06656390 clinical trial (Ruan et al., 2026), clinical activities and safety profiles of other anti-FGFR2b ADCs are also anticipated. In contrast, current trend toward bispecific ADCs (Katoh et al., 2025) warrants development of investigational FGFR2b x CLDN18.2, FGFR2b x EGFR, FGFR2b x HER2 and FGFR2b x MET bispecific ADCs (Figure 1).

## 4 Common mechanisms of resistance to biologics

Limited anti-tumor cytotoxicity in the immunosuppressive TME harboring exhausted CD8^+^ T cells, angiogenic endothelial cells, fibrotic cancer-associated fibroblasts (CAFs) and other suppressor cells (Davidson et al., 2021; Huinen et al., 2021; Katoh and Katoh, 2022) as well as TAA/epitope loss in cancer cells owing to intra/intertumor heterogeneity and isoform switching (Katoh et al., 2025) are common mechanisms of resistance to TAA-targeting biologics (Bradley and Anczuków, 2023; Chang et al., 2023; Lamplugh et al., 2026; van de Donk et al., 2024), such as mAbs, ADCs, bsAbs and CAR-T cells (Figure 4). It is noteworthy that multiple mechanisms, such as (1) immune exhaustion impairing cytotoxic T-cell functions, (2) fibrosis preventing the access of immune cells and biologics and (3) biologics-degrading acidic microenvironment, elicit resistance to biologics in the immunosuppressive TME.

**FIGURE 4:**
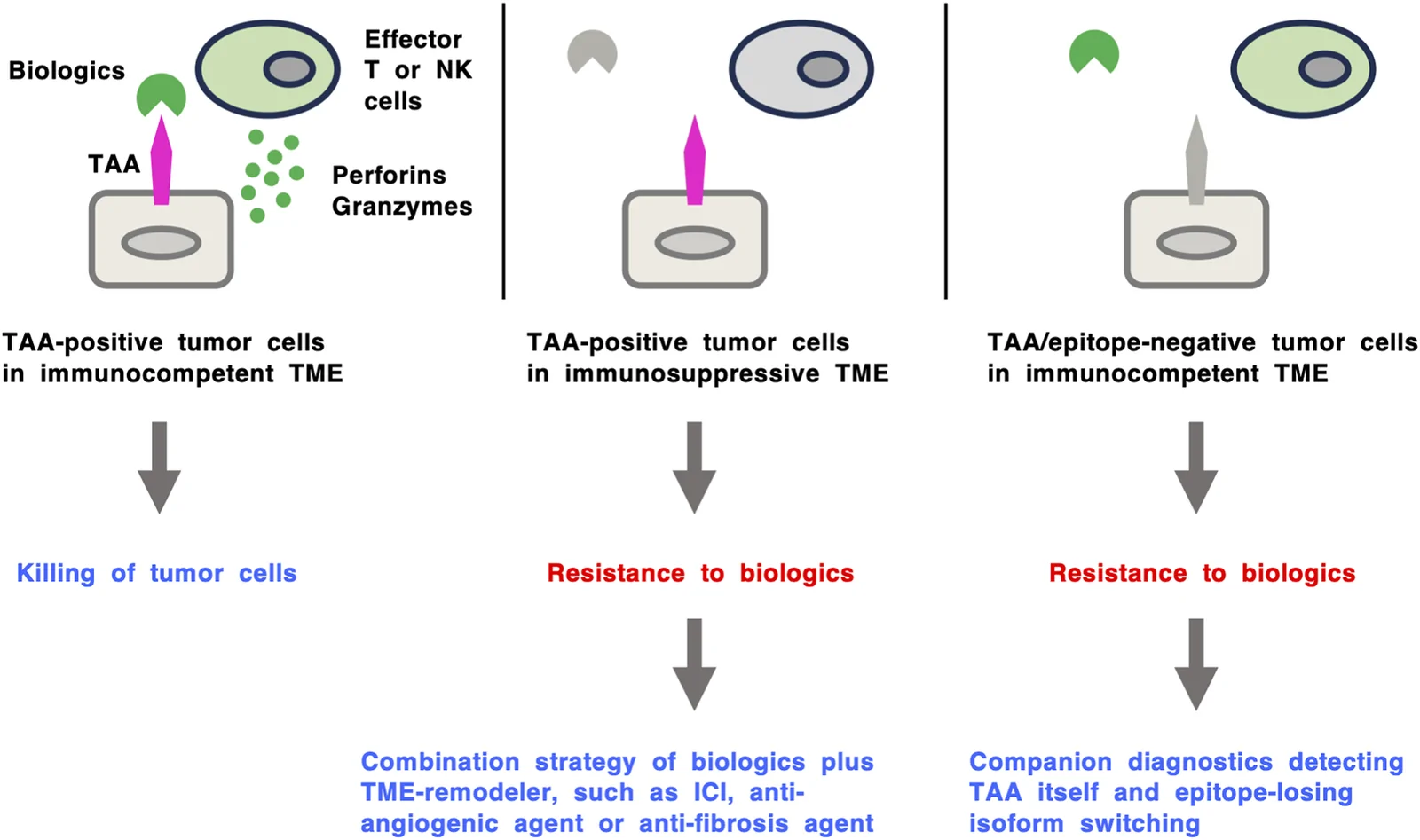
Response and resistance to biologics, such as monoclonal antibody, antibody–drug conjugate, bispecific antibody and chimeric antigen receptor T cells. (Left) Tumor-associated antigen (TAA)-positive tumor cells in the immunocompetent tumor microenvironment (TME) are recognized by effector T cells and natural killer (NK) cells, which induce cytotoxicity via secretion of perforins and granzymes. (Middle) TAA-positive tumor cells in immunosuppressive TME are resistant to biologics as a result of degeneration/exclusion of biologics or exhaustion/exclusion of immune cells. (Left) TAA/epitope-negative tumor cells in immunocompetent TME are also resistant to biologics owing to the escape from immune surveillance. Combination strategy of biologics plus TME remodeler, such as immune-checkpoint inhibitor (ICI) or anti-angiogenic/fibrosis agent, is anticipated to mitigate biologics resistance in the immunosuppressive TME. In contrast, companion diagnostics detecting TAA isoforms in primary and metastatic lesions are necessary to predict biologics resistance owing to TAA/epitope loss associated with therapy-induced transdifferentiation of tumor cells.

Combination strategies of TAA-targeted biologics plus TME remodeling agents, such as ICI, anti-angiogenic agents and CAF-targeted drugs, are anticipated to improve clinical activities of biologics in the immunosuppressive TME, because TME remodelers convert immunosuppressive TME into immunocompetent TME in preclinical studies (Davidson et al., 2021; Huinen et al., 2021; Katoh and Katoh, 2022). Among these combination strategies, clinical trials of ICI-based combination therapies with CLDN18.2-targeted mAbs (Table 1), ADCs (Table 2), bsAbs (Table 3) and CAR–T cells (Table 4) are ongoing.

In contrast, CLDN18.2 and FGFR2b splicing variants can be switched into other CLDNs and FGFR2c owing to therapy-induced transdifferentiation and epithelial–mesenchymal transition, respectively, in cancer patients (Katoh et al., 2024b). Clinical development of companion diagnostics detecting TAA itself and TAA isoforms in the primary and metastatic lesions using IHC or nucleic acid-based technologies (Armstrong et al., 2019; Sengal et al., 2021; Yashiro et al., 2021) are warranted to monitor TAA/epitope loss and predict resistance to biologics in the clinical settings (Katoh et al., 2025).

## 5 Conclusion

Splice variant-targeted biologics have opened up new avenues for cancer therapy because some splice variants are *bona fide* TAAs, regardless of their functions as cancer drivers or passengers. Zolbetuximab and bemarituzumab are pioneering mAbs targeting CLDN18.2 and FGFR2b splicing variants, respectively, while next-generation biologics targeting these TAAs in the form of ADCs, bsAbs and CAR-T cells are emerging with expectations for improved clinical activities and mitigated adverse events. Because immune evasion and epitope-losing isoform switching are common mechanisms of resistance to biologics, the clinical development of ICI-based combination strategies and diagnostics methods for detecting alternative isoforms are also anticipated. In contrast, cost-effectiveness and quality of life are critical issues for patients and supporting family members as well as governments and health insurance systems. Comparative studies on ADC monotherapy versus mAb/chemo-combination therapy and the clinical development of bispecific ADCs and allogeneic CAR-T cells should further improve the clinical benefits of CLDN18.2- or FGFR2b-targeted therapies.
